# Advancement in pre- and peri-procedural imaging

**DOI:** 10.1093/eurheartjsupp/suaf095

**Published:** 2026-03-05

**Authors:** Georg Nickenig, Marcel Weber, Rebecca T Hahn, Marta Sitges, Julien Dreyfus

**Affiliations:** Heart Center, Medical Clinic II, University Hospital Bonn, Venusberg-Campus 1, Bonn 53127, Germany; Heart Center, Medical Clinic II, University Hospital Bonn, Venusberg-Campus 1, Bonn 53127, Germany; Department of Medicine, Division of Cardiology, Irving Medical Center, Columbia University, New York, New York, USA; Cardiovascular Institute, Hospital Clinic, IDIBAPS, CIBER Cardiovascular, University of Barcelona, Barcelona, Spain; Cardiology Department, Centre Cardiologique du Nord, Saint-Denis, France

**Keywords:** Tricuspid regurgitation, Imaging

## Abstract

Only recently, pathophysiology and relevance of tricuspid regurgitation (TR) has been acknowledged but remains still poorly understood. The rising interest in this valve disease has also fuelled the efforts to define and improve imaging modalities. Vice versa, understanding tricuspid disease initiation and progress, validation, and quantification as well as choice and monitoring of treatment critically rely on reproducible and sensitive imaging capabilities. The latter is dominated by ever improving ultrasound-based analysis applied from the outside, the oesophagus or within the heart. The increasing appreciation of the relevance of TR and the rapid evolution of catheter-based treatment options has stimulated engineers and industry to swiftly improve established methodologies as well as to provide novel tools including three-dimensional intra-cardiac echocardiography and fusion imaging. Ultrasound is necessarily complemented by MRI and CT in the tricuspid space urging the cardiologist to gain also expertise in these modalities. In this review, we will focus on the quantitative and reproducible measurements to quantify TR and concomitant left and right heart anatomy, function, and haemodynamics before, during and after treatment. In addition, we will share insights on adjunct and future technologies.

## Routine and advanced imaging modalities

Imaging support is essential for the safe and effective implementation of transcatheter treatments for tricuspid regurgitation (TR), as well as for the appropriate selection of candidates for these procedures and for patient follow-up. Several imaging techniques are used to guide this process (*Table 1*). Routine imaging of the tricuspid valve (TV) includes transthoracic and transoesophageal echocardiography (TTE). Advanced imaging with multi-planar reconstructions from 3D TTE and cardiac computed tomography (CT) can be applied to more accurately define TV anatomy and plan transcatheter procedures. Advanced imaging also includes cardiac magnetic resonance (CMR) to evaluate the hemodynamic impact of TR.

**Table 1:** Main advantages and limitations of cardiac imaging techniques used before and during transcatheter interventions for the tricuspid valve

Modality	Applications	Main advantages	Main limitations
Transthoracic echocardiography (TTE)	First-line diagnosis; assessment of TR severity, RV size/function	Widely available, non-invasive, provides hemodynamic data and initial anatomical evaluation	Limited acoustic windows; lower resolution, especially for TV leaflet evaluation
Transoesophageal echocardiography (TEE)	Detailed TV anatomy; intra-procedural guidance (repair/replacement)	High resolution; critical for leaflet motion and coaptation; flow imaging; real-time guiding	Sub-optimal alignment with TV; technically challenging due to leaflet thinness and anterior location; accoustic shadowing from left heart devices and interatrial septum
Cardiac magnetic resonance (CMR)	Assessment of RV volumes and systolic function	Most accurate for RV function and volume; prognostic value; complementary to echo	Not used during procedures; unclear cut-off for futile intervention. Limited availability
Cardiac computed tomography (CT)	Pre-procedural planning, especially for TV replacement and annular-based repairs	Excellent anatomical detail; gold standard for annulus sizing and RCA proximity; useful for lead relation and venous access planning	Exposure to radiation and contrast; not used intra-procedurally
3D intra-cardiac echocardiography (3D-ICE)	Intra-procedural guidance, especially when TEE is sub-optimal	Real-time guiding; bypasses TEE limitations; avoids oesophageal route	Lower image quality than TEE; high cost; limited availability
Fusion imaging	Enhances procedural navigation by combining modalities (e.g. CT + echo + fluoroscopy)	Real-time overlay; improves spatial orientation and device placement. Shortens learning curve	Not widely available; workflow integration still evolving and still time consuming

### Transthoracic echocardiography

Transthoracic echocardiography (TTE) is the first-line test for diagnosing and evaluating TR. It enables detection of the presence of regurgitation and helps to assess its severity using a grading scale (typically five grades) and an integrative approach,^1^ which is key for identifying potential candidates for transcatheter treatment.

**Figure 1:**
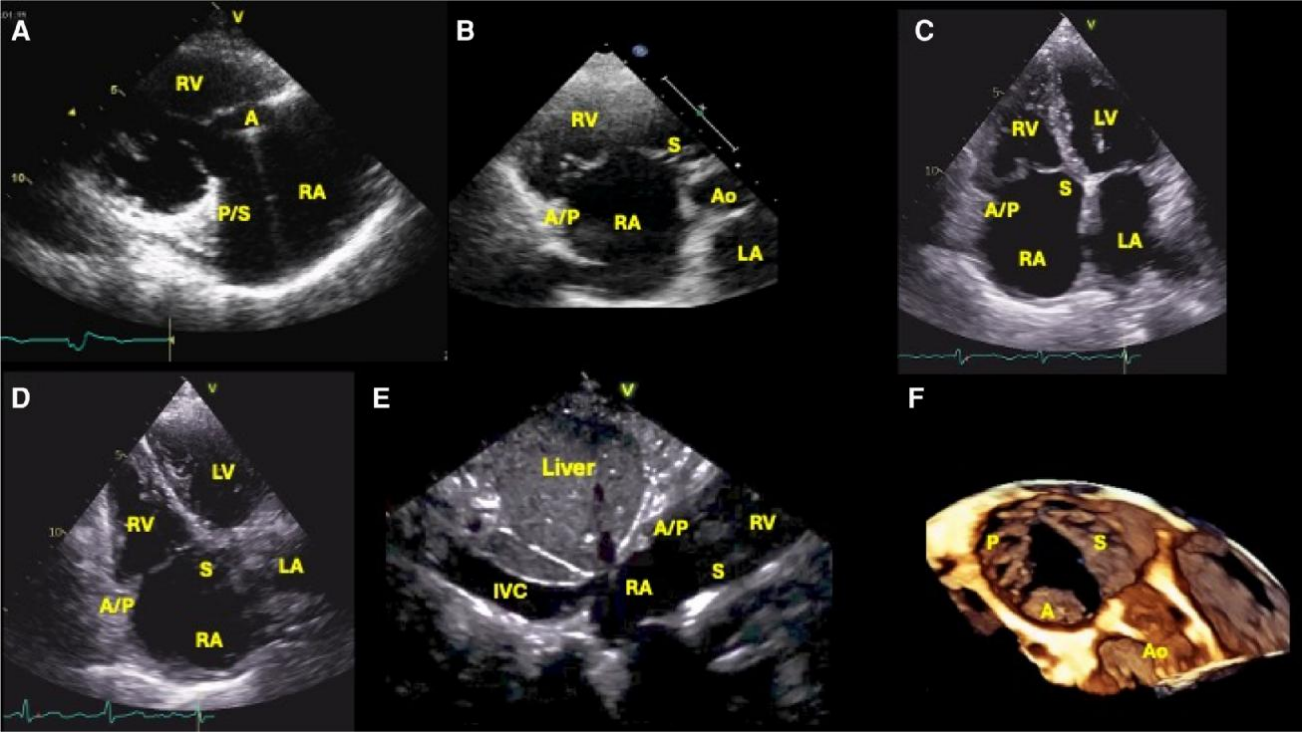
Systematic TTE for the tricuspid valve: essential views. (*A*) Long parasternal axis depicting RV inflow; (*B*) parasternal short-axis view at the level of the great vessels; (*C*) conventional four chamber apical view; (*D*) right side focused four chamber apical view; (*E*) sub-costal long-axis view focused on the right atria and the inferior vena cava; (*F*) 3D en-face view of the TV from the right atria. A, anterior leaflet of the tricuspid valve; Ao, aortic valve; IVC, inferior vena cava; LA, left atrium; LV, left ventricle; P, posterior leaflet of the tricuspid valve; RA, right atrium; RV, right ventricle; S, septal leaflet of the tricuspid valve.

TTE also provides initial information on the size and function of the right ventricle (RV),^2^ the geometry of the right atrium (RA), and any left-sided cardiac pathology. Combined with the patient's clinical history, this helps determine the aetiology and phenotype of TR. It is particularly important to evaluate the hemodynamic impact of TR on RV dilation and function, as well as the dynamics of the inferior vena cava—easily assessed from a sub-costal view. These are important factors to select the most appropriate timing and type of intervention. TTE can also give insights into the movement and coaptation of the TV leaflets, which is especially relevant in patients with intra-cardiac leads. An essential systematic algorithm of the TV with TTE should include a parasternal inflow view of the RV obtained by tilting the face of the probe towards the diaphragm from a standard long-axis parasternal view (*Figure 1A*), a short-axis view at the level of the great vessels (*Figure 1B*), a 4-chamber apical view with probe centred on the LV apex (*Figure 1C*), an RV-focused view with probe over the lateral LV apex to optimize RV and RV dimensions (*Figure 1D*) and finally the inferior vena cava evaluation from the sub-costal view (*Figure 1E*). Three-dimensional (3D) en-face views of the TV can be also obtained in patients with good acoustic window, comprehensively depicting the complete anatomy (commissures and leaflets) of the TV (*Figure 1F*).

### Transoesophageal echocardiography

**Figure 2:**
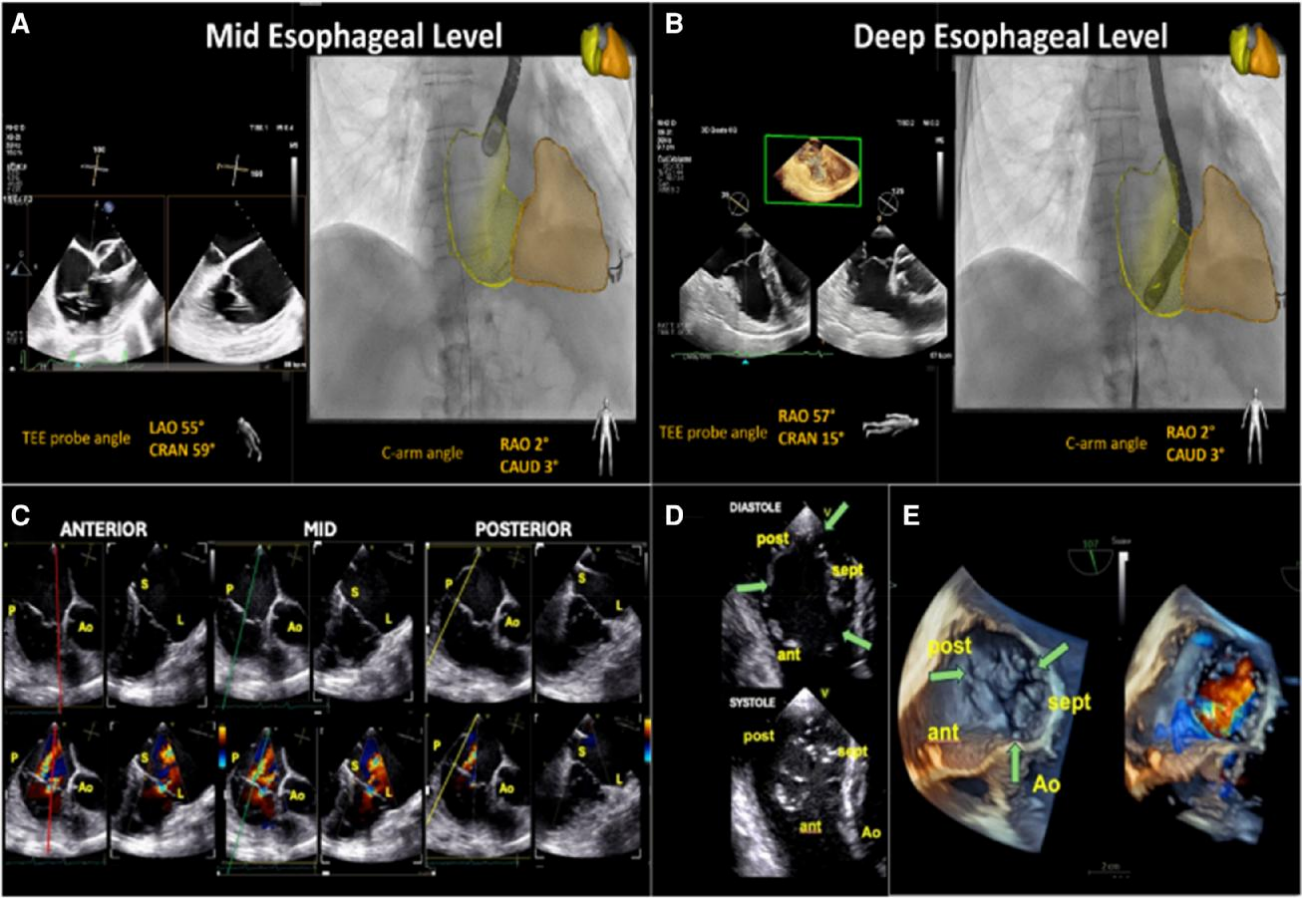
Key transoesophageal echocardiographic views for tricuspid valve assessment. (*A*) RV inflow–outflow view with simultaneous orthogonal views (Xplane, Biplane) depicting the RV inflow (right side focused four chamber view), acquired using a three-dimensional TEE probe at the mid-oesophageal level with the corresponding C-arm angulation and its superimposed projection on the angiography. (*B*) RV inflow view (right side focused four chamber view) with simultaneous orthogonal views (Xplane, Biplane) depicting the RV inflow–outflow view, acquired using a three-dimensional TEE probe at the deep-oesophageal level with the corresponding C-arm angulation and its superimposed projection on the angiography. (*C*) At the mid-oesophageal level, the three panels represent progressive slices of the tricuspid valve from anterior (red line, left panel), central (green line, middle panel), to posterior (yellow line, right panel), with the latter being the most distant from the aortic valve and therefore, posterior. Colour Doppler imaging in each panel enables comprehensive functional assessment, including leaflet motion, leaflet tethering, leaflet length, and identification of the primary origin of tricuspid regurgitant flow. AO, aortic valve (anterior); L, lateral; P, posterior; S, septal. (*D*) Short-axis transgastric view and 3D en-face view of the tricuspid valve. Left panel: Short-axis view of the tricuspid valve obtained from the transgastric level. The image depicts the tricuspid valve in diastole with three leaflets and three commissures indicated by green arrows (upper panel), alongside the same valve in systole (lower panel). Ant, anterior; AO, aortic valve (anterior); Post, posterior; Sept, septal. (*E*) Three-dimensional en-face TEE view from the right atrial perspective, oriented similarly to the transgastric short-axis view, with the aortic valve positioned at 5 o’clock on a clock-face orientation. The three leaflets and corresponding commissures (green arrows) are clearly visible. The right panel demonstrates the same 3D view with colour Doppler imaging, highlighting the central origin of tricuspid regurgitant flow between the coaptation surfaces of the anterior and septal leaflets. Ant, anterior; AO, aortic valve (anterior); Post, posterior; Sept, septal.

TEE is an instrumental tool for advanced diagnosis and detailed anatomical and functional assessment of the TV. TEE offers higher-resolution imaging of the TV leaflets and sub-valvular apparatus. However, imaging the TV with TEE can be challenging due to its anterior and right-sided location and the consequent far field and sub-optimal alignment with the probe within the oesophagus. In addition, the leaflets of the TV are thinner and variable in number, making them more difficult to visualize by echocardiography. Despite these limitations, TEE remains the most critical imaging technique for detailed TV assessment and for guiding transcatheter TV interventions, including both repair and replacement procedures. Essential views to evaluate the TV are shown in *Figure 2*. The RV inflow–outflow view obtained at the mid- or deep-oesophageal level with simultaneous orthogonal views (Xplane, biplane) (*Figure 2A* and *B*), allows for a comprehensive assessment of the anterior and posterior leaflet coaptation with the septal leaflet (*Figure 2C*). The short-axis view of the TV obtained in the transgastric plane (*Figure 2D*) and the 3D en-face view of the TV acquired from any imaging plane (*Figure 2E*) are essential for assessing leaflet number/morphology, coaptation gaps and location of the regurgitant jet.

**Figure 3:**
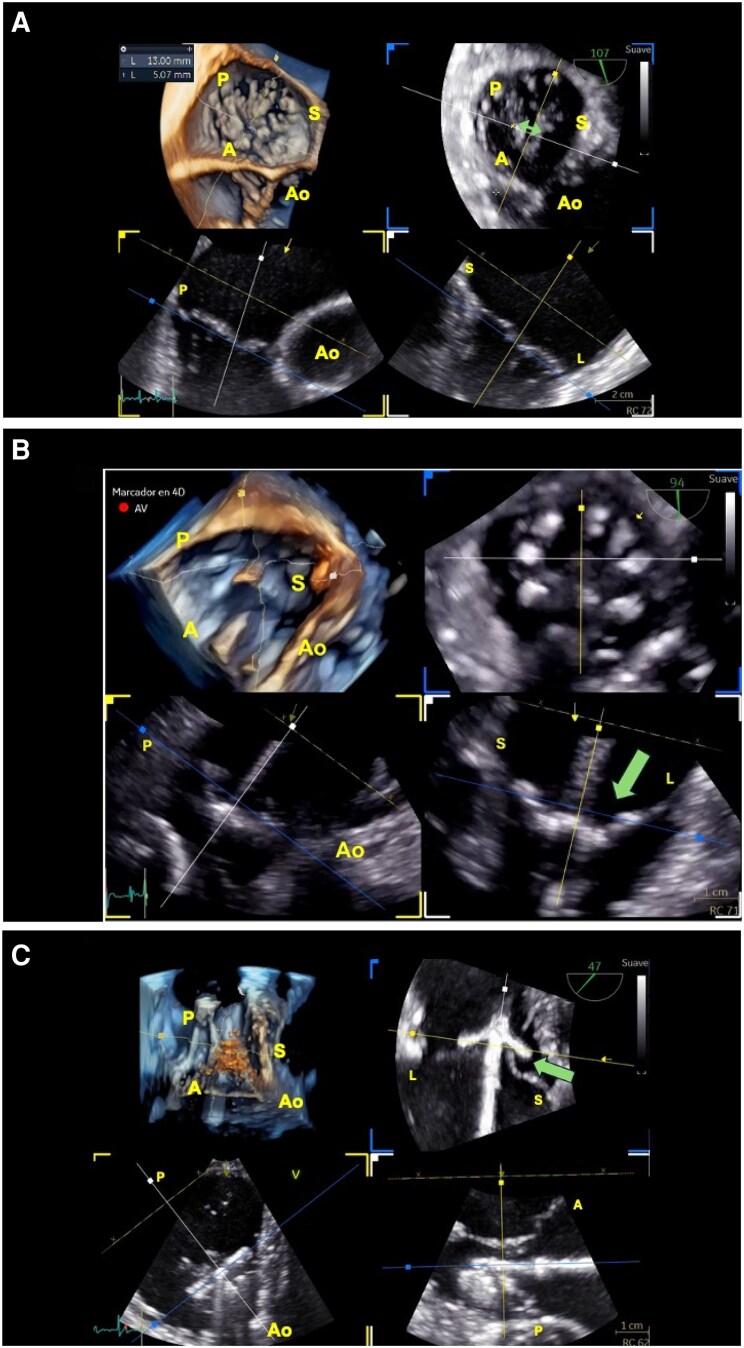
Multi-planar reconstruction of the tricuspid valve using advanced three-dimensional transoesophageal echocardiography. (*A*) The tricuspid valve is displayed in a short-axis view from the right atrial perspective, with the aortic valve oriented at the 5 o’clock position in a clock-face format (upper left panel). Two orthogonal imaging planes are shown: one aligned with the right ventricular inflow–outflow axis (yellow line, yellow square on left lower panel), depicting the anteroposterior dimensions of the tricuspid valve, and a second plane orthogonal to the first (indicated by the white line and white box in lower right panel), demonstrating the septolateral dimension of the valve. The blue line and box (upper right panel) depicts a short-axis view of the valve at the level of the leaflets tips allowing for the precise measurement of the coaptation gap (green arrow), accurately located with this type of reconstruction. A, anterior; AO, aortic valve (anterior); L, lateral; P, posterior; S, septal. (*B*) Real-time three-dimensional echocardiographic (3D TEE) multi-parametric reconstruction during transcatheter tricuspid valve implantation (Evoque, Edwards Lifesciences). The image illustrates the use of real-time multi-planar reconstruction (MPR) to align the imaging planes with the trajectory of the delivery catheter (White and yellow lines and boxes, lower panels). The alignment confirms a coaxial trajectory with the annular valve plane and accurate central positioning of the prosthesis at the level of the tricuspid annulus. This technique also enables by cutting the image at the levels if the leaftlets and prosthesis anchors (blue line and box, upper right panel), a helical rotation around the anchoring zones of the prosthetic valve, facilitating assessment of proper engagement of the native tricuspid leaflets into the prosthetic anchors. Optimal leaflet capture ensures secure anchoring and effective device deployment. AO, aortic valve (anterior); L, lateral; P, posterior; S, septal. (*C*) Multi-planar reconstruction derived from a three-dimensional transoesophageal echocardiographic (3D TEE) dataset acquired in a transgastric short-axis view during transcatheter edge-to-edge tricuspid valve repair. The reconstruction allows precise alignment with the trajectory of the delivery catheter and optimal orientation of the repair device. This facilitates confirmation of coaxiality with the valvular plane, accurate device positioning, and appropriate depth of catheter advancement into the right ventricle. Simultaneous visualization of both septolateral and anteroposterior planes enables detailed assessment of device-leaflet interaction, ensuring effective capture of the tricuspid leaflets and procedural success. A, anterior; AO, aortic valve (anterior); L, lateral; P, posterior; S, septal.

TEE plays a pivotal role in the guidance of transcatheter interventions on the TV, confirming the diagnosis of TR, RV function status, and most critically, allowing for a comprehensive assessment of the functional anatomy of the TV. This assessment includes detailed visualization of the number and spatial arrangement of the TV leaflets, identification of commissures/indentations, evaluation of leaflet motion and tethering as well as measurement of the TV annular dimensions. Importantly, TEE allows for assessment of the interaction between intra-cardiac leads and the TV apparatus (i.e. leaflet, chordae, or papillary muscles), aiding in determining whether the leads contribute to the aetiology of TR. A thorough understanding of the patient-specific functional anatomy of the TV and the underlying pathophysiological mechanisms responsible for the regurgitation is essential for selecting the most appropriate therapeutic strategy. These aspects must be evaluated during the selection process in patients that may be considered for a transcatheter TV intervention. Multi-planar imaging derived from 3D TEE acquisitions allow for this detailed examination and are also essential during procedure guidance (*Figure 3A–C*).

### CMR imaging

**Figure 4:**
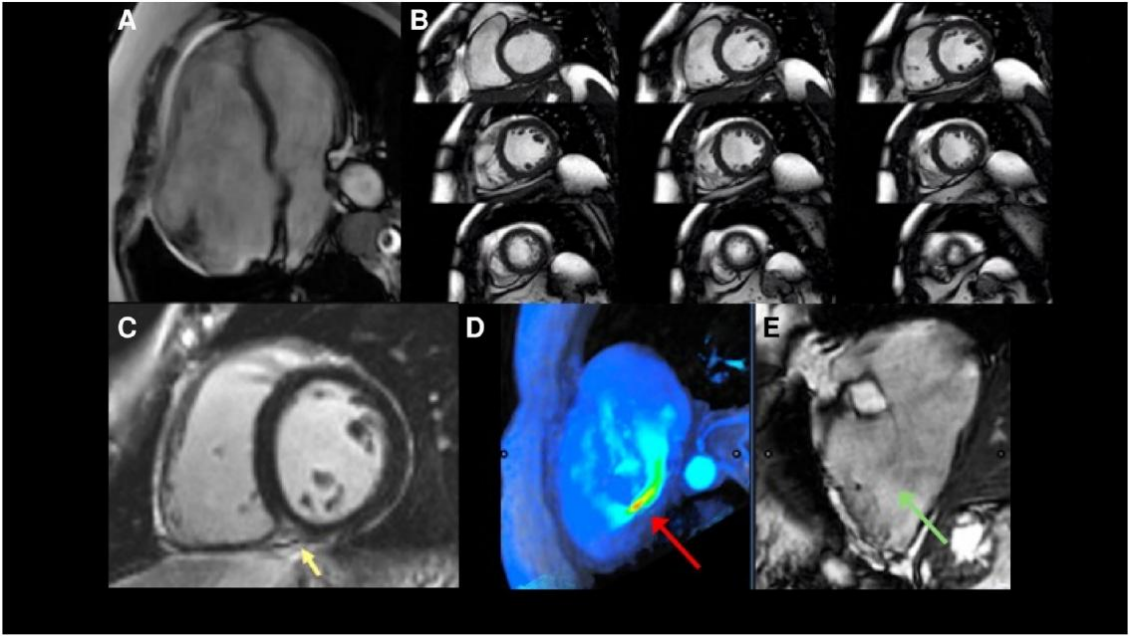
Key contributions of cardiac magnetic resonance in tricuspid regurgitation evaluation. (*A*) Four chamber cine sequence depicting a dilated right ventricle (RV); (*B*) short-axis cine sequences used to delineate the ventricular endocardial border to estimate RV volumen and function; (*C*) late contrast acquisition to characterize RV tissue depicting late enhancement in the interventricular septum (yellow arrow); this corresponds to myocardial fibrosis, typically observed in RV overload; (*D*) and (*E*) 4D Flow acquisition across the tricuspid valve demonstrating a tricuspid regurgitant Flow (panel *D*, red arrow) with a clear Flow convergence at the level of the point of the gap of leaflet coaptation in the corresponding cine sequence (panel *E*, green arrow).

CMR imaging is a highly accurate tool for assessing right heart chamber volumes and RV systolic function as well as quantify TR regurgitant volume and regurgitant fraction (*Figure 4*).^3^ This information is crucial for identifying appropriate candidates for transcatheter TV therapies. Although CMR plays no intra-procedural role, it provides unique and complementary data on RV dilation and functional status. Recent studies have proposed a RV end-diastolic volume larger than 150 mL/m^2^ BSA as a potential poor prognostic marker in patients with TR, suggesting that this could be a potential parameter to indicate intervention. However, the precise point at which RV dysfunction becomes severe enough to render any form of intervention futile remains undefined.

The role of stress CMR or stress echocardiography in assessing RV contractile reserve is not yet well defined.^4^ This assessment could potentially be useful in identifying patients who may still benefit from transcatheter intervention despite borderline RV function. Finally, CMR may be used following transcatheter device therapies to assess changes in chamber sizes and function.^5^

### Cardiac CT

**Figure 5:**
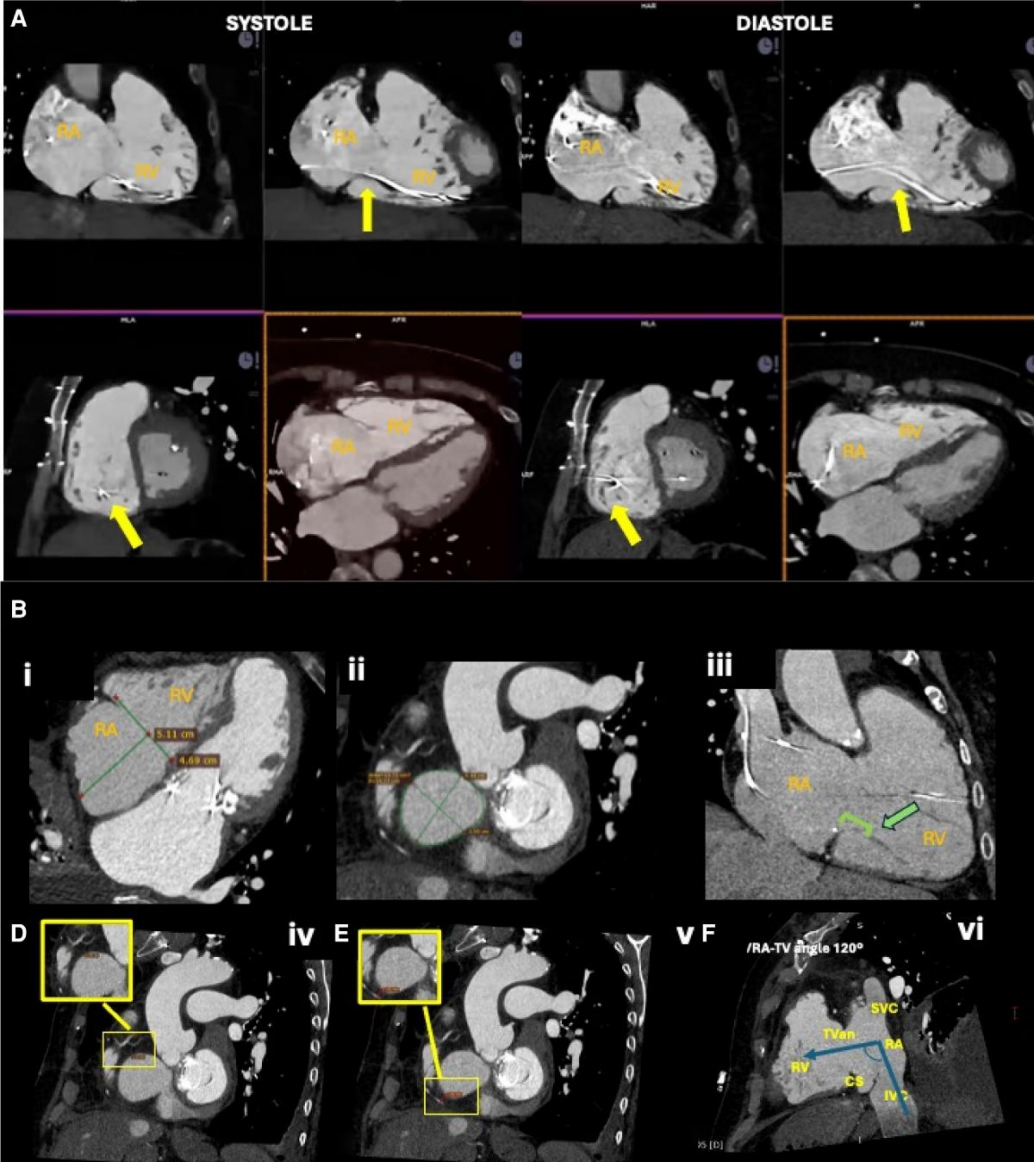
Cardiac computed tomography (CT) images. (*A*) Cardiac computed tomography (CT) images illustrating the relationship between an implantable cardioverter-defibrillator (ICD) lead and the tricuspid valve apparatus. The systolic image demonstrates close contact between the lead and the tricuspid leaflets, while the diastolic image shows the leaflets fully separated and independent from the lead (yellow arrow), indicating that the lead is mobile and does not interfere with tricuspid valve function. The high spatial resolution of CT imaging allows for detailed and precise assessment of the lead’s position relative to the motion of the leaflets and the sub-valvular tricuspid apparatus. RA, right atrium; RV, right ventricle. (*B*) Role of cardiac computed tomography (CT) in the assessment of the tricuspid valve. (i) Measurement of left atrial size and tricuspid annular dimensions. (ii) Measurement of the tricuspid valve annulus. (iii) Evaluation of the tricuspid sub-valvular apparatus, including its relationship with the leaflets and the distance from the papillary muscles to the tricuspid annulus (green arrow). (iv and v) Measurement of the distance from the tricuspid annulus to the right coronary artery at the anterior (panel iv) and posterior (panel v) annular segments. (vi) Assessment of the inferior vena cava (IVC) entry into the right atrium and the projected angulation required to achieve coaxial alignment with the tricuspid annular plane. CS, coronary sinus; IVC, inferior vena cava; RA, right atrium; RV, right ventricle; SVC, superior vena cava; TV an, tricuspid valve annulus.

Cardiac CT is essential for procedural planning, particularly in the context of transcatheter TV replacement and, in selected cases, for tricuspid repair, especially when using annular-based devices.^6^ CT imaging provides detailed information on right heart chamber volumes and high-resolution anatomical visualization of the TV, including the number of scallops, leaflet indentations, and, importantly, the spatial relationship of the valve leaflets to existing ventricular leads (*Figure 5A*). It is also the gold-standard modality for determining the dimensions of the TA, which is critical for appropriate sizing and selection of transcatheter valve prostheses and the proximity of the right coronary annulus. Furthermore, CT allows for precise evaluation of the entry points of the superior and inferior venae cavae into the RA, aiding in the prediction of catheter trajectory and optimal fluoroscopic projection during device implantation. *Figure 5B* illustrates the role of CT.

### Three-dimensional intra-cardiac echocardiography

Three-dimensional intra-cardiac echocardiography (3D-ICE) catheters are emerging as valuable tools for real-time, high-resolution imaging of the TV from within the RA.^7^ These devices offer 3D and multi-planar reconstruction capabilities, overcoming many of the imaging limitations associated with TEE for tricuspid interventions such as shadowing from lipomatous interatrial septum or mechanical prosthesis in the left heart. Current image quality is limited by the current size and shape of the catheter, which thus require probe manipulation to optimize the use of the long-axis of the probe (thus using the greatest number of piezoelectric elements). These probes have proven highly useful, particularly for transcatheter TV repair procedures.^7^ Main limiting factors for its routine use include limited availability and especially, high costs as these catheters are currently not reusable.

### Multi-modality imaging fusion

Significant efforts are underway to improve the precision and safety of transcatheter TV procedures through advanced imaging integration. Image fusion—particularly the real-time overlay of fluoroscopy and echocardiography—is already part of routine practice. Additionally, novel software solutions are under development to allow real-time fusion of multiple imaging modalities, including CT, echocardiography, and angiography. Preliminary experiences have demonstrated the potential of combining CT with echocardiography or angiography to enhance anatomical localization and procedural guidance.^8,9^ However, these techniques still remain largely investigational and are not yet widely adopted in daily clinical practice.

## How to diagnose severity of TR

### Characterizing TR severity

**Figure 6:**
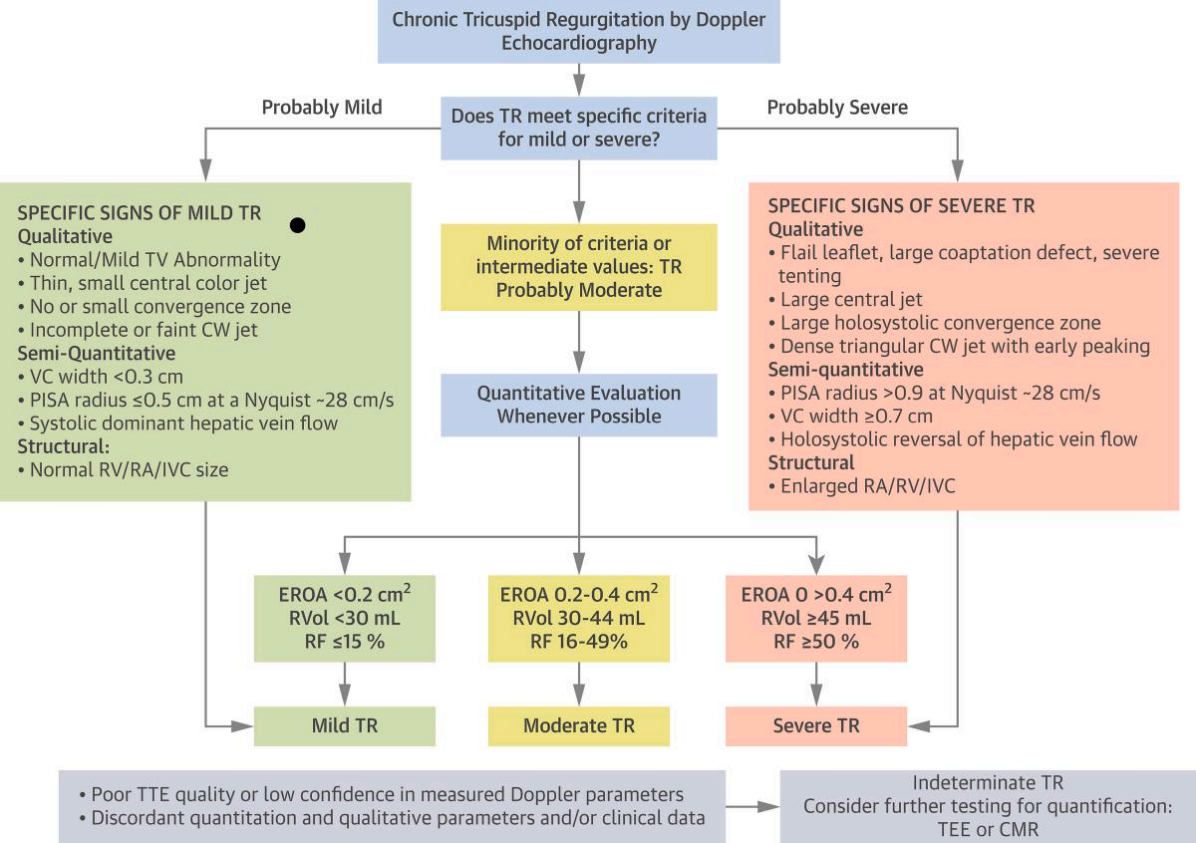
Algorithm for the integration of multiple echocardiographic parameters of TR severity (reprinted from^15^, with permission from Elsevier). CW, continuous wave; EROA, effective regurgitant orifice area; IVC, inferior vena cava; PISA, proximal isovelocity surface area; RA, right atrium; RV, right ventricle; RVOL, regurgitation volume; TR, tricuspid regurgitation; TV, tricuspid valve; VC, vena contracta.

Although standard TR assessment by Doppler echocardiography is based on grading the TR severity as mild, moderate and severe, the extended grades of massive and torrential are associated with adverse outcomes^10–12^ and are recommended for grading severity in patients being considered for transcatheter tricuspid valve interventions (TTVI).^1,13^ Various structural, qualitative, semi-quantitative, and quantitative criteria may be integrated for the purpose of grade definition (*Figure 6*).^14^ Some criteria are considered specific of mild TR (e.g. small central colour jet or systolic dominant hepatic vein flow) or of severe TR (e.g. incomplete tricuspid coaptation or systolic reversal of hepatic vein flow), with moderate TR designated when signs are mixed/lacking.

**Figure 7:**
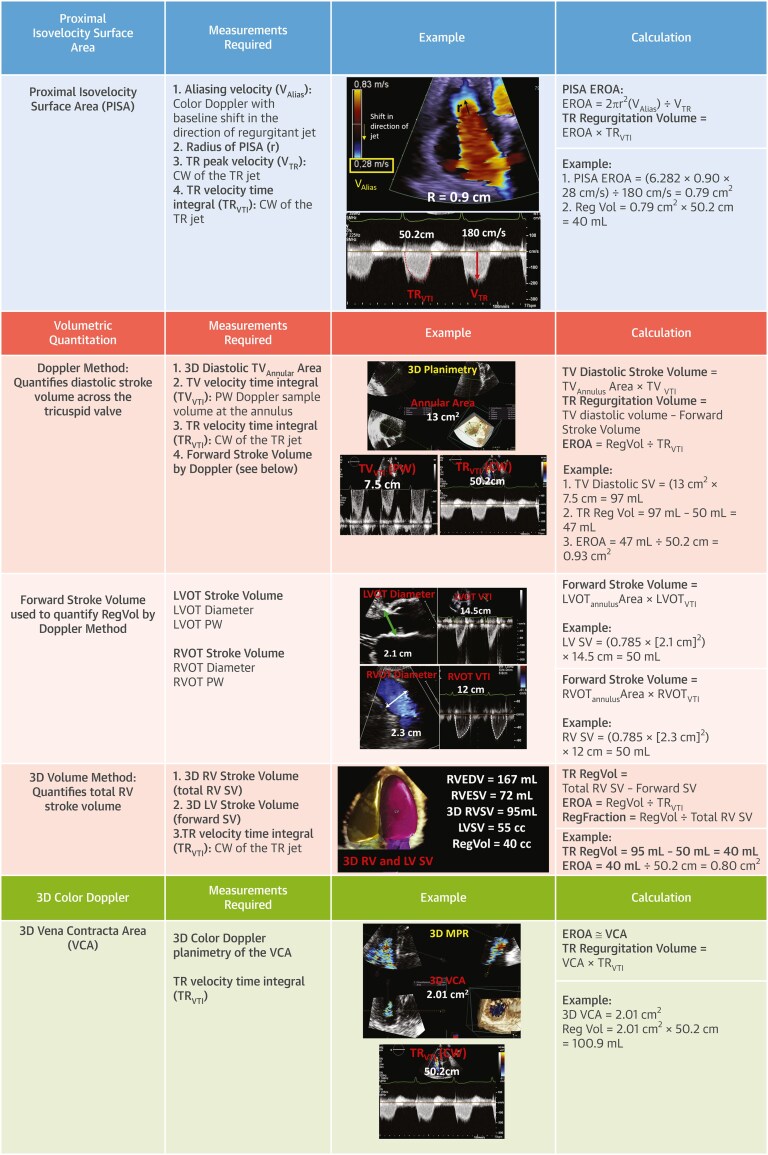
Standard and novel methods to quantify TR (reprinted from^16^, with permission from Elsevier). CW, continuous wave; EROA, effective regurgitant orifice area; IVC, inferior vena cava; LVOT, left ventricular outflow tract; PISA, proximal isovelocity surface area; RA, right atrium; RV, right ventricle; RVOL, regurgitation volume; RVOT, right ventricular outflow tract; TR, tricuspid regurgitation; TV, tricuspid valve; VC, vena contracta.

Guidelines suggest that quantifying TR should be performed, in order to reduce the variability of assessment using a multi-parametric, integrative approach. These methods include: proximal isovelocity surface area (PISA) measurement of effective regurgitant orifice area (EROA), direct measurement of 3-dimensional vena contracta area (3D-VCA) from 3D colour Doppler datasets, and volumetric quantitation, which subtracts forward stroke volume (SV) from the total diastolic SV to quantify regurgitant volume (*Figure 7*).

**Figure 8:**
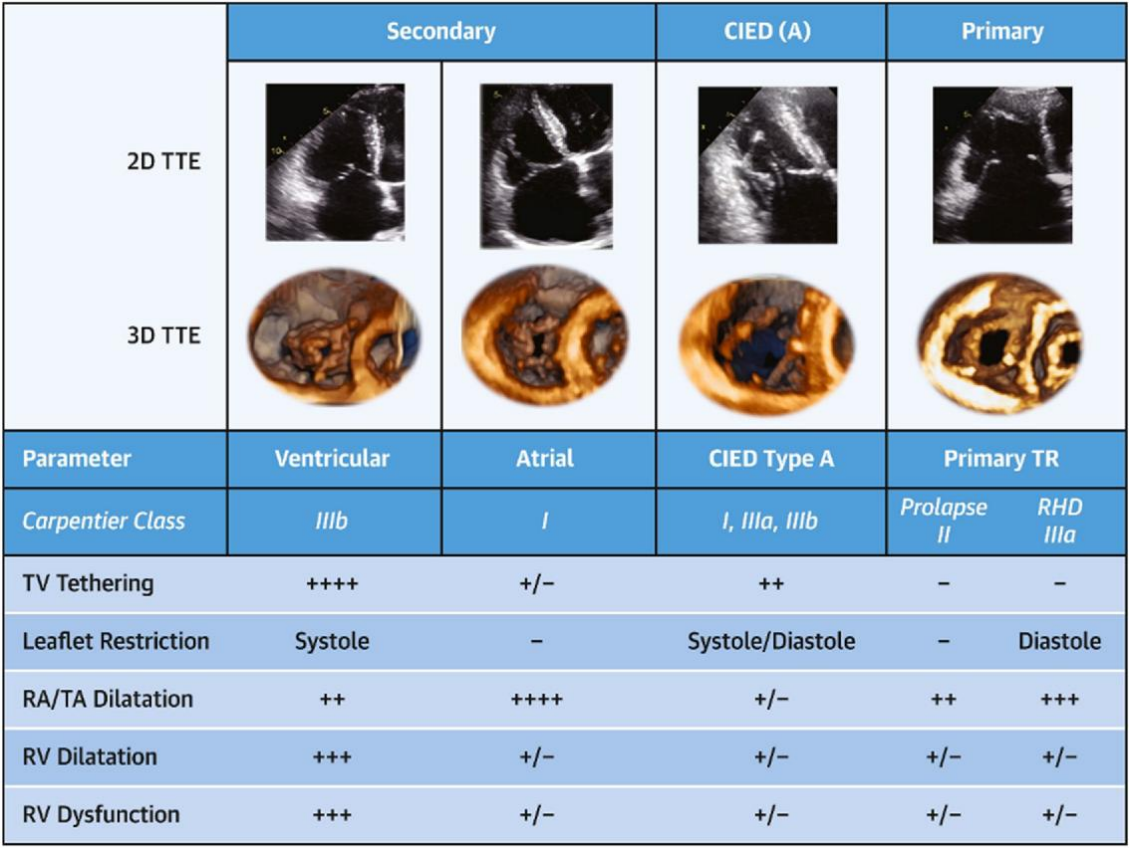
Classification of TR aetiology (reprinted from^18^, with permission from Elsevier). The expanded tricuspid regurgitation (TR) classification by aetiology currently separates secondary TR into atrial secondary and ventricular secondary disease. When cardiac implantable electronic device (CIED) leads are present, lead-associated TR may be sub-categorized into type A, whose CIED is causing the TR, and type B, whose CIED is incidental. 2D, 2-dimensional; 3D, 3-dimensional; RA, right atrium; RV, right ventricle; TA, tricuspid annulus; TTE, transthoracic echocardiography; RHD, rheumatic heart disease; TV, tricuspid valve.

Characterizing TR severity also requires determining TR aetiology and the associated findings of these different causes of leaflet malcoaptation. The classification proposed by the PCR tricuspid focus group^17^ and the Tricuspid Valve Academic Research Consortium^18^ stratifies the TR into primary, secondary atrial, secondary ventricular, and related to a cardiac implantable electronic device (CIED) (*Figure 8*). Primary TR is due to direct valvular involvement, such as prolapse, flail, carcinoid, endocarditis, rheumatic or congenital abnormalities. In secondary TR (STR), valve leaflets are structurally normal and regurgitation results either from the predominant dilation and dysfunction of: (i) the right RA—atrial STR, due to atrial fibrillation (AF) or less frequently heart failure with preserved ejection fraction, or (ii) the RV—ventricular STR, often related to left heart disease or pulmonary hypertension.^19^ CIED-related TR refers to TV dysfunction caused by pacemaker or defibrillator leads, which can interfere with leaflet mobility either by direct mechanical impingement or entanglement of any part of the valve apparatus, by causing structural damage to the valve, leading to significant regurgitation, or pacing-induced RV dysfunction.^20^

Because some of these different aetiologies are associated with differing risks for mortality^21,22^ it is important to assess chamber sizes, left and right ventricular function, and pulmonary artery pressures in order to determine these aetiologies. However, the assessment of features such as coaptation gaps, leaflet tethering, chamber sizes, and function, may all play a role in device choice as well.

### Assessing the TV for TTVI

**Figure 9:**
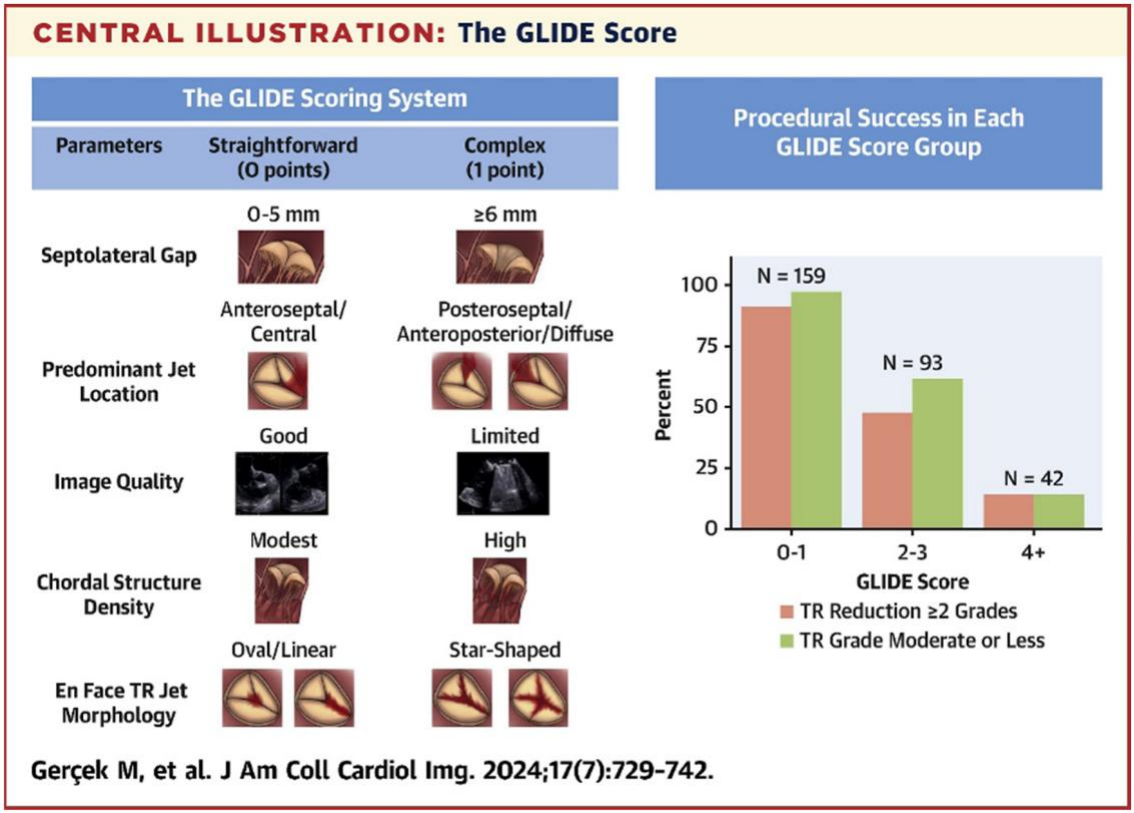
The GLIDE score. Points were assigned to five features which have predicted residual tricuspid regurgitation following T-TEER: septolateral coaptation gap, TR jet location, chordal structure density, image quality, and en-face TV morphology. A GLIDE (**G**ap, **L**ocation, **I**mage quality, **D**ensity of chordae, **e**n-face morphology) score of 0–1 was associated with acceptable technical outcomes (TR reduction ≥2 grades and ≤ moderate TR) and correlated with improvement in NYHA functional class and 6-minute walk distance at 3 months. Illustration was reused from.^24^

Multi-modality imaging plays a crucial role in assessing the TV anatomy, TR aetiology, and severity.^14,23^ In addition, anatomic features may also be integral to the prediction of procedural success by evaluating the entire TV apparatus, right heart structure, and adjacent structures.^24,25^ The multiple identified predictors of residual TR following transcatheter interventions are summarized in *Table 2*. Simplified predictive scores for residual TR following transcatheter edge-to-edge repair (TEER), the GLIDE score (gap, location, image quality, density of chordae, and en-face morphology) assessed the risk based on only five features (septolateral coaptation gap, TR jet location, chordal structure density, image quality, and en-face TV morphology).^24^ A score of 0–1 was associated with acceptable technical outcomes (TR reduction ≥2 grades and ≤moderate TR) and correlated with improvement in NYHA functional class and 6-minute walk distance at 3 months (*Figure 9*).

**Table 2:** Predictors of residual TR following transcatheter interventions (modified from^15^)

Anatomy of the tricuspid valve and sub-valvular apparatus
Presence of multiple indentations or leaflets and chordal complexity are associated with less TR reduction for T-TEER^26^ but may not be a predictor of technical success with other devices (i.e. direct annuloplasty^27^ and TTVR.^28,29^ High leaflet-to-annulus ratio (<1.06) predicts ≥3+ TR following T-TEER.^30^ Sub-valvular anatomy (i.e. papillary muscle number, location, or height) may impact TTVR device delivery.^28^ Tenting area >1.92 cm^2^ and tenting height ≥10 mm associated with residual TR for T-TEER.^31,32^ Atrial secondary TR may not be a determinant of T-TEER efficacy.^33,34^
Jet location, coaptation gap, and TR severity
Non-anteroseptal jet location is associated with lower technical success^35^ and may impact the improvement in cardiac output^36^ for the T-TEER devices. Large coaptation gap (>8 mm) is predictors of less effective TR reduction after T-TEER^32,35,37–39^ and direct annuloplasty.^40^ Greater than severe TR grade (i.e. massive or torrential) is associated with lower technical success for T-TEER^11,12,31,32^ but is not a consideration for TTVR which reduces TR similarly for all baseline grades of TR.^41^
Right heart anatomy
Larger RA and RV size may reduce procedural and technical device success for T-TEER.^32,35,42^ Larger RV dimensions have been associated with worse outcomes after T-TEER.^35,42^ Small RV length can be prohibitive for TTVR valve implantation.^28,43^ Specific parameters for device-specific anchoring: Angle of the interventricular septum relative to the tricuspid annular plane (ideally 80° to 100°) facilitates septal anchoring for the LUX valve.^44^
CIED involvement in TR genesis
CIED-related TR, including adhesion, sub-valvular entanglement, or perforation, favours surgical treatment or TTVR.^20,45^ Risks of jailing leads should be considered. T-TEER can also be considered with incidental CIED leads, or if the location of the regurgitant jet allows device implantation,^46^ however the presence of a CIED is predictive of > moderate residual TR.^32^
Caval vein anatomy
Inferior vena cava offset in patients with severe TR^47^ is associated with procedural complexity during T-TEER.^42^ Inferior or superior vena caval offset may affect implantation of orthotopic TTVR.^28^ Superior and inferior vena cavae dimensions, and angulation^48^ as well as supracardiac veins (jugularis, azygos, and brachiocephalic) and distance from the inferior vena cava orifice to the hepatic vein, are assess to avoid occlusion bicaval valve implantation (e.g. TricValve).
Procedural image quality
High resolution intra-procedural imaging is essential for all transcatheter TV devices. Repair devices typically require higher imaging resolution and multiple imaging levels/windows compared with TTVR, to visualize leaflet pathology and adequacy of leaflet insertion for the T-TEER devices, and anchor placement and insertion for the direct annuloplasty devices.^15^ 3D intra-cardiac echocardiography (ICE) can be considered to provide complementary information and optimize procedural success.^7^

## Intra-procedural TEE imaging for transcatheter TV interventions

TEE is the cornerstone imaging modality during TTVI, providing real-time visualization of the TV anatomy, device positioning, and immediate results. This section outlines key TEE imaging protocols during two categories of catheter-based TV interventions: edge-to-edge repair and valve replacement.

### TEE views and protocols

Standard TEE perspectives for the TV are shown in *Figure 2*. The mid-oesophageal (ME) RV inflow–outflow view (at ∼70°) serves as a ‘commissural’ view of the TV, displaying the anterior and posterior leaflets with the septal leaflet out of plane.^49^ Using this as a primary view, simultaneous biplane imaging can sweep from right to left across the septal leaflet coaptation line from the anteroseptal to posteroseptal commissures (reversed four chamber or ‘grasping’ view), helping localize regurgitant jets (*Figure 2C*). Deep oesophageal views allow imaging through right-heart structures alone (avoiding shadowing from left-heart devices). Transgastric (TG) views (with anteflexion usually at 30°) provide an en-face short-axis of the tricuspid orifice, visualizing all three leaflets and the origin of TR jets. This TG view is essential for aligning device orientation (e.g. perpendicular alignment of repair devices’ grasping arms) and assessing coaptation gaps. Biplane imaging (30° + 120°) even allows more information of device depths and localization of cordae as well as papillary muscles. To image trajectory, position and orientation of the device in real time, MPR views can be used to image 2D orthogonal long-axis views of the device for trajectory, as well as 2D and 3D short-axis views for position and orientation.

Pre-procedural planning should confirm adequate TEE windows in a supine position; patients in whom leaflets cannot be visualized clearly are poor candidates for TEE-guided repair.

### Edge-to-edge repair (leaflet approximation)

TEER is by far the most common TV intervention worldwide. Devices like the TriClip (Abbott)—a dedicated tricuspid variant of the MitraClip—and the PASCAL repair system (Edwards Lifesciences) grasp and coapt the native leaflets, mimicking the surgical edge-to-edge (Alfieri) stitch.

### Imaging workflow

**Figure 10:**
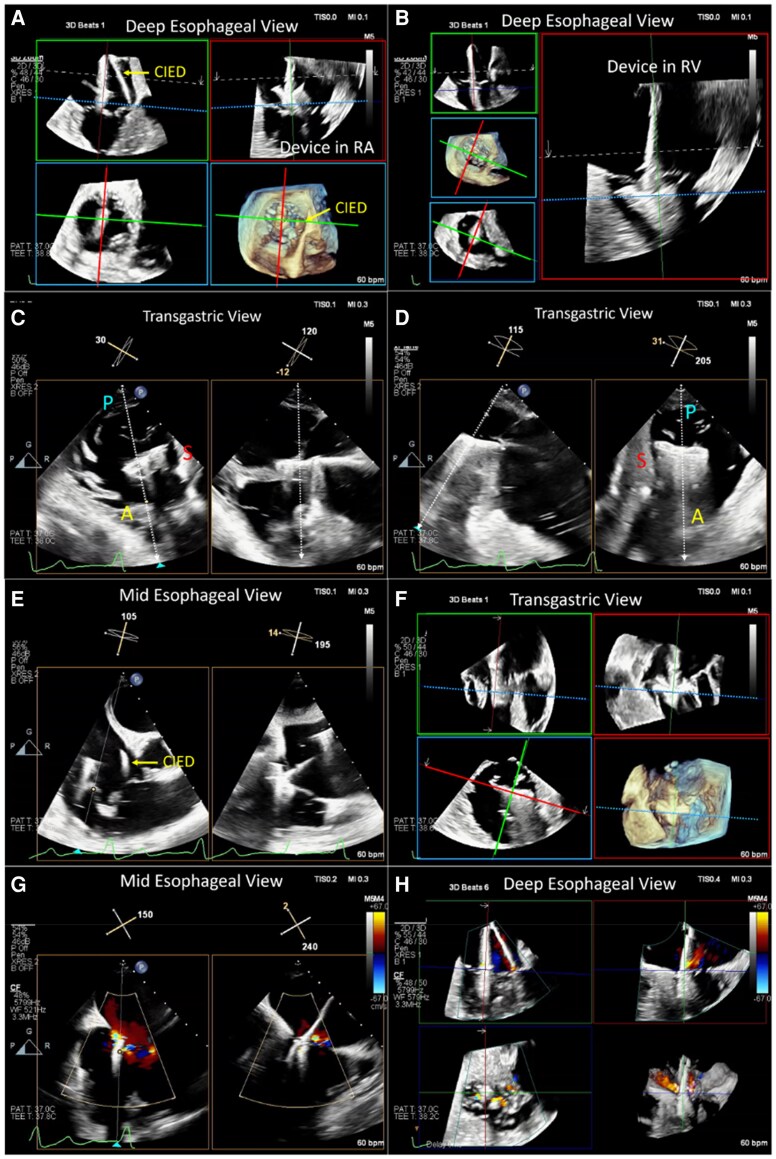
Standard intra-procedural TEE views. Left: 60° ME RV inflow–outflow view + XPLANE with colour (150°, reversed four chamber view). Right: TG view at 40° with XPLANE at 130°. XImaging modalities and levels for TEER. Imaging for tricuspid TEER can be performed from any imaging level (mid-oesophageal, deep oesophageal, and transgastric views. Using multi-planar reconstruction (MPR, panel *A* and *B*) is useful for confirming trajectory relative to the annular plane (blue dotted line) from orthogonal views (red and green planes), as well as position and orientation from short-axis images (2D and 3D blue planes). Once the device is below the leaflets, a transgastric view should be used to confirm orientation. Panel (*C*) shows a biplane image using the short-axis view (SAX) as the primary image, however if the device if the leaflet tips are not perfectly aligned in the SAX view (∼30°), an inflow–outflow view (∼115°) can be used understanding that the orthogonal SAX view is now reversed (septal leaflet on the left). If 3D imaging is not adequate for imaging leaflet grasp, 2D single plane or biplane (panel *C*) imaging can also be used to improve temporal and spatial resolution. Because transgastric views (panel *D*) may image leaflets and chordae better than oesophageal views, this view can also be used for leaflet grasp; importantly, confirmation of leaflet grasp is most accurate using MPR aligned with the device arms (red panels). Following closure of the device, residual TR is assessed by 2D and 3D colour Doppler (panels *E* and *F*, respectively). Note: this patient had a cardiac implantable electronic device lead (yellow arrow) in the anterior-septal commissure.

Intra-procedural TEE is crucial during all phases of TEER. Initially, with the guide catheter and clip delivery system entering the RA, TEE in a bicaval view confirms the catheter location and steering towards the valve plane. The ME RV inflow–outflow (‘commissural’) view at ∼60° as well as the XPLANE image at ∼150° (‘grasping view’) is then used to direct the device towards the TR jet origin (*Figure 10A*). Colour Doppler in this view helps target the principal regurgitant orifice along the septal leaflet length. Once the TEER device is centred over the regurgitant jet, the next task is to align the delivery catheter perpendicular to the line of leaflet coaptation. If this succeeds grippers/sliders are tested to facilitate independent grasping. Continuous imaging of the device being advanced into the RV is required since changes in position and orientation may occur depending on the trajectory achieved (*Figure 10B*). After that the TEE TG short-axis view is employed to verify that the device arms are oriented perpendicular to the line of leaflet coaptation. This en-face view ensures the device is well orientated and will grasp the correct leaflet segments (usually the anterior and septal leaflets for central jets, or septal and posterior leaflets for posteroseptal jets) (*Figure 10C*).

**Figure 11:**
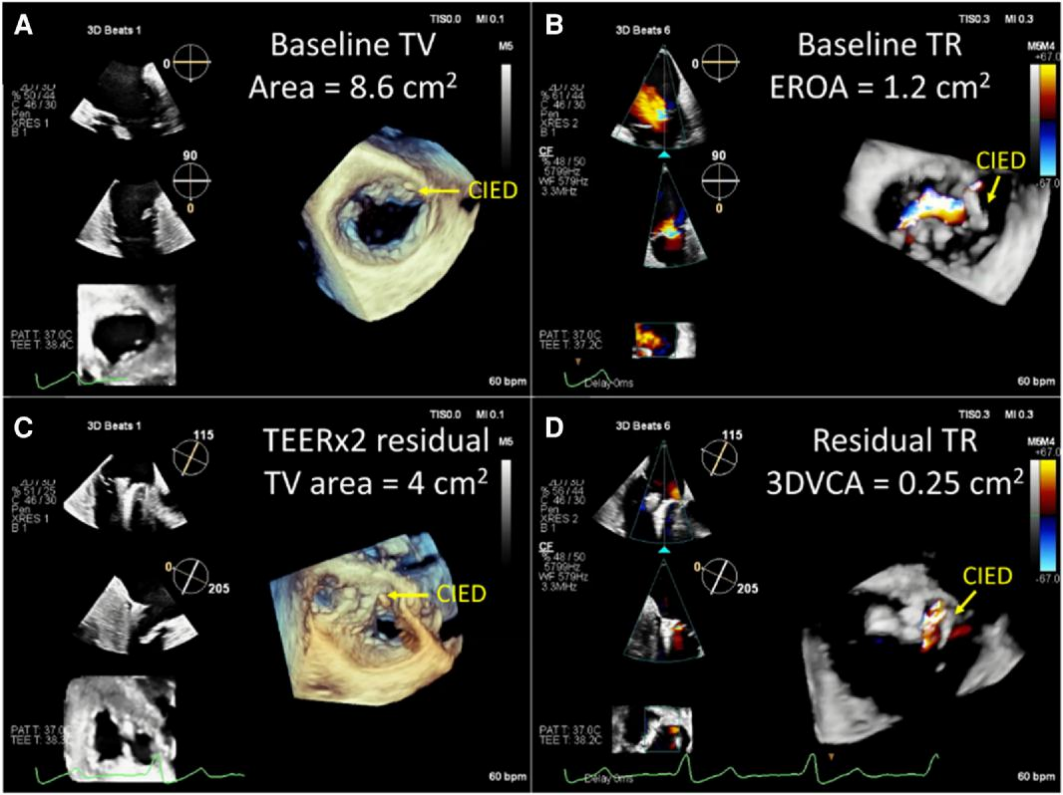
Post-TEER assessment. Compared with baseline imaging (panels *A* and *B*), implantation of 2 TEER devices resulted in a residual tricuspid valve (TV) area of 4.0 cm^2^ (panel *C*) and residual TR by three-dimensional planimetry of the vena contracta area (3D-VCA) of 25 mm^2^ consistent with mild disease.

During leaflet grasping, simultaneous multi-plane/Xplane imaging may yield the highest temporal and spatial resolution, although if 3D multi-planar reconstructions (MPR) images are adequate, real-time assessment of trajectory, position, and orientation can also be used to assess leaflet grasp (*Figure 10B*). If leaflet insertion is unclear, 3D zoomed en-face views or MPR can help verify both leaflets are engaged before device closure. Grasping view (mid-TEE at 140–160°) is usually good for visualizing both leaflets (*Figure 11*), while the TG view is useful for orienting and positioning the clip.^50^ If the TG view is clear, the short-axis view is just as effective for grasping. During leaflet grasping, switch between both views to ensure that the culprit lesion of TR is targeted and that sufficient leaflet tissue is captured within both clip arms.^51^ Before deployment of a clip, colour Doppler assessments of residual TR by measuring the vena contracta and PISA as well as the measurement of mean diastolic *trans*-tricuspid gradients are mandatory. The imaging team must confirm that no significant stenosis (tricuspid mean pressure gradient >3 mmHg) has been induced.

### Transcatheter tricuspid valve replacement

Replacing the TV with a transcatheter bioprosthetic valve is an appealing option for patients with severe TR, particularly those for whom repair is not feasible. Early experiences have used NaviGate GATE valves (NaviGate Cardiac Structures), Evoque (Edwards Lifesciences), Topaz (Tricares), CardioValve (Venus Medtech), and LuxValve (Jenscare) Prostheses. Up to now, the Evoque prosthesis is the only one to have obtained CE mark and FDA approval.

**Figure 12:**
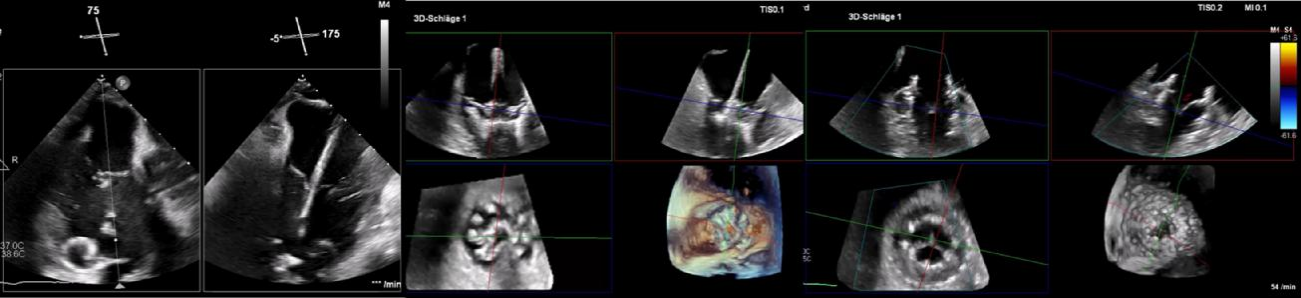
Intra-procedural imaging of TTVR with the Evoque prosthesis. Left: 75° ‘commissural view’ showing extra small Safari wire in RV and biplane with 175° displaying perpendicular trajectory of Evoque delivery system. Middle: Live MPR imaging of ventricular grasping legs visualized in three different planes while loading leaflets. Right: MPR colour imaging of deployed Evoque prosthesis in systole.

For these transcatheter tricuspid valve replacement (TTVR) devices, imaging windows and planes are quite similar to TEER procedures. While a pigtail pre-shaped support guidewire is delivered via a steerable sheath, TEE confirms wire position in a RV-focused view at 0°, in a grasping view (ME TEE at 140–160°) or a long-axis TG view (*Figure 12*). Important is that the wire is not entangled in chordae or trabeculations and is positioned posterior to the anterior papillary muscle. When the support wire is positioned correctly the delivery system is advanced to the TV. Similar to TEER, trajectory and positioning is key for procedural success. For this step, the ME RV inflow–outflow (‘commissural’) view at 60° as well as the XPLANE image at 150° (‘grasping view’) and the TG long-axis view (120–150°) are essential. Additionally, device location, depth, and trajectory are optimized using 3D MPR imaging from mid- or deep-oesophageal windows (*Figure 12*). While most TTVR prostheses rely on ventricular grasping legs or arms, as well as an atrial plate that mimics a sandwich mechanism, the primary task of the procedure is to make sure that the amount of ventricular grasping arms catches tricuspid leaflets properly. This is verified in the commissural and grasping view using simultaneous biplane imaging as well as MPR imaging from mid- or deep-oesophageal windows. When a proper grasp of most of the leaflet's circumference has been achieved, the half-opened prosthesis is elevated to the atrium and the atrial plate is released. This sandwich mechanism ensures a proper seating of the device. After valve deployment, transvalvular and paravalvular regurgitation is assessed and mean gradients are measured^28^ (*Figure 12*).

Intra-procedural TEE imaging is fundamental to the success of catheter-based tricuspid therapies. By employing a systematic multi-plane approach, from ME commissural views to TG en-face views, the imaging team can guide devices with precision in the challenging right-heart environment.

## Imaging after TTVr and TTVR

Imaging post-TV intervention device should include a comprehensive evaluation of device position/stability and function, as well as a complete assessment of the size and function of right and left heart cardiac chamber.^18^ Changes in right and left heart size and function as well as changes in cardiac output should be part of the routine follow-up assessment. Novel measures of RV function include the assessment of effective RV ejection fraction forward SV divided by total RV end-diastolic volume^52,53^ as well as right ventricular-to-pulmonary artery (RV-PA) coupling assessed by forward SV divided by RV end-systolic volume.^54^

Whereas, echocardiography remains the first line imaging modality, multi-modality imaging plays an important role as well. Both CMR and CT can assess right-sided reverse remodelling after TV intervention by quantifying the changes in RA and RV volumes and RV contractile function,^5^ as well as a positive increase in left ventricle (LV) volumes due to improvement of pre-procedural chronic LV underfilling.Moreover, in case of TEER, CT has been used to evaluate the orientation of the TEER devices, single leaflet device attachment and residual anatomical regurgitant orifice area. TTE or TEE suspicion or evidence for thrombus (i.e. visualized thrombus/leaflet thickening or increased transvalvular gradient) should be confirmed by CT which can detect hypo-attenuating leaflet thickening.

Patients undergoing TV interventions present unique challenges for quantifying residual TR: (i) lack of validation for quantitative parameters of TR severity following device implantation; (ii) specific device-related challenges of colour Doppler evaluation (more likely after T-TEER with multiple, non-coaxial jets with proximal flow restricted by the device).^55^ Therefore, the approach to the post-procedural evaluation post-device TR severity is currently to integrate multiple parameters rather than emphasize or depend on a single measurement; this helps mitigate the effects of technical or measurement errors inherent to each method.^56^ Although the 3D-VCA method could be more appropriate for quantitation or multiple and/or irregular regurgitant orifices, it is not well validated and multi-beat acquisitions may be required to optimize temporal and spatial resolution. Normalization of the hepatic venous flow pattern would point to a significant reduction in TR severity with the caveat that late systolic flow reversal may be seen in the setting of a non-compliant RA.^57^ Beyond the challenges in quantifying residual TR, the objectives and cut-off values for each parameter remain ill-defined following transcatheter treatment, particularly as the need for greater granularity compared with the currently proposed grading system has been suggested.^55,58^

## Future imaging techniques and modalities

### Technological change and artificial intelligence

Imaging is shaping the field of structural heart interventions and especially in the mitral and tricuspid space.^15^ Since live 3D TEE became commercially available in the early 2000s, structural interventions have developed magnificently. Following this, the temporal and spatial resolution of the echo images improved by a factor of at least 10. Currently, live 3D acquisition is capable to acquire 40–50 volumes per second without and 10–20 volumes per second with colour Doppler.^59^ New developments of ultrafast ultrasound can sample up to 2325 volumes per second at a depth of 12 cm, which will greatly improve spatial and temporal resolution.^60^ There is no question that the three largest companies providing hard- and software for TTE and TTE (GE Vingmed, Philips and Siemens Healthineers) will continue to significantly improve image quality over the next years.

Same applies to CMR and CT. Higher resolution and frame rate enables better assessment of RV SV and pulmonary artery forward flow measurements in CMR. More precise anatomical measurements and procedural planning for TTVR be significantly improved by refining CT slice thickness from the current standard of 0.5–0.75 mm down towards 0.2–0.3 mm. This advancement will be made possible by the introduction of faster dual-source CT scanners and the latest generation of photon-counting CT systems.^61^ In addition to further development of proven technology, artificial intelligence-supported heart valve diagnostics is set to improve the quality of examinations and help even inexperienced doctors perform accurate echocardiography. The ability to integrate and process large amounts of data simultaneously in order to identify information that might be missed by the human eye is particularly appealing.^62^ This may lead to greater awareness of heart valve disease and these AI tools have been proposed to guide less experienced users. Consequently, fewer patients may be misdiagnosed. For example, machine learning models have emerged in the field of TR, providing a more accurate prediction of RV-PA coupling by estimating invasive mean pulmonary arterial pressure from echocardiographic parameters.^63^ In combination with echocardiography, advances in deep learning for interpreting cardiac CT and CMR images could help to standardize the process, leading to greater accuracy in TR grading and RV function assessment. These developments will further improve transcatheter valve interventions.

### Robotic TEE

To prevent interventional echocardiographers from radiation and to achieve consistent high-quality imaging, robotic TEE promises improvement. First-in-human experience using a novel remote-controlled robotic system developed by ROB’E for remotely manipulating a TEE probe were reported.^64^ In this study, all standard cardiac views were successfully replicated using the robot with 100% clinical and technical success and no complications. Although robotic control took slightly longer (average 12 min vs. 9 min for manual), it demonstrated feasibility and safety for real-time imaging guidance.

### 3D- ICE

Early experience demonstrated feasibility of using ICE as the sole imaging modality or complement to TEE in performing transcatheter TV repair.^65,66^ Recently, two 3D-ICE catheters have entered the European market: the Philips VeriSight Pro and the Siemens AcuNav Lumos Catheter. A third 3D-ICE system (Johnson & Johnson NUVISION) is already available in the US but has not yet received CE mark. As these are first-generation imaging catheters, we expect improvements in both the hardware and software in the future. The enhancements will deliver better image resolution and streamlined, automated workflows, facilitating more straightforward and consistent imaging guidance during structural heart procedures.^7^ In Europe, reimbursement for intra-cardiac imaging is inadequate. This may delay the adoption of 3D-ICE as a standard clinical procedure. In the future, it will be important to improve image quality, sector width, and frame rate in order to fully exploit the advantage of the small distance between the probe and the valve to be repaired. If 3D-ICE can completely replace TEE in TV interventions, it has the potential to eliminate the need for anaesthesia in patients, thereby reducing procedure time and increasing patient safety.^67^

### Fusion imaging

The fusion imaging technique can acquire imaging data from different sources, primarily fluoroscopic and TEE images, and align these images in three dimensions and over time. So far, few human studies have evaluated combining echocardiography and fluoroscopy for transcatheter TV repair or replacement. The technique can simplify catheter navigation and potentially reduce fluoroscopy use.^68^ While fusion imaging has reduced procedural times in some structural heart interventions (e.g. trans-septal puncture), evidence in TV interventions is limited to small series, and no improvements in short- or long-term outcomes have been demonstrated to date.^69^

**Figure 13:**
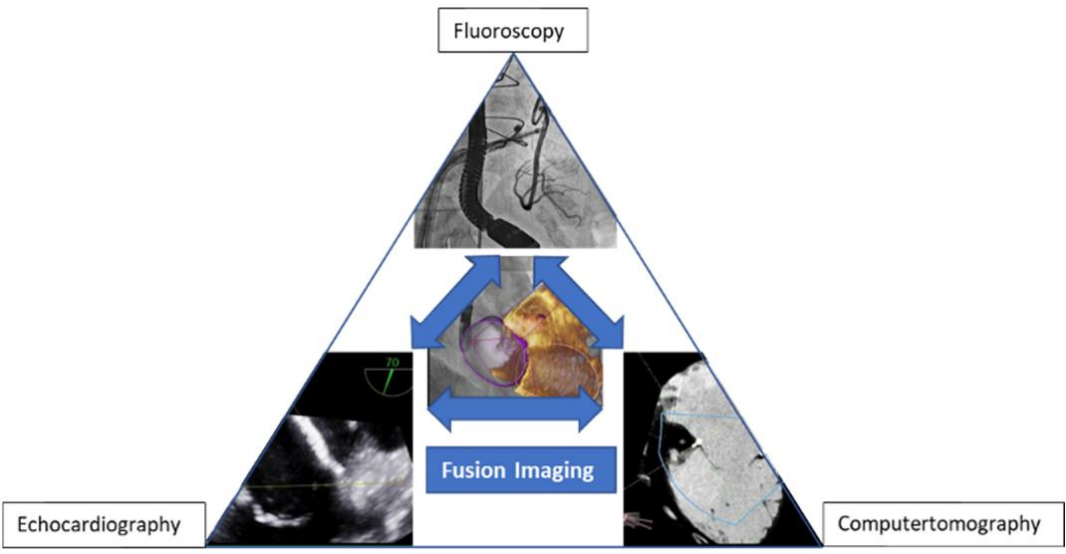
Fusion from different image modalities. Illustration of echocardiography, computed tomography and fluoroscopy with their fusion possibilities.

The simultaneous fusion of all three image modalities (echocardiography, CT, and fluoroscopy) would be desirable for the future (*Figure 13*). This could expedite the learning curve of structural interventions and make complex catheter procedures easier and safer for the patient.

### 3D printing and virtual reality

Finally, cardiac 3D printing is an emerging technology that enables the production of patient-specific cardiac anatomical replicas based on imaging data. It has already been explored as part of the pre-procedural planning process for transcatheter interventions, providing a realistic simulation.^70^ This is particularly true for heart surgery, where it can facilitate pre-operative planning and training for complex procedures. The widespread use of this technique is hampered by a lack of standardization and their cost. This novel technique could swiftly become outdated due to the emergence of cutting-edge augmented reality technologies, but haptic feedback of 3D printing models will remain a unique feature, particularly for surgical training purposes.

## Conclusions

Quality and progress of interventional cardiology is tightly and essentially coupled to the advancements in imaging techniques. Feasibility of a catheter-based procedure relies besides patients and anatomy characteristics on imaging quality. In addition, efficacy, predictability, reproducibility, ease-of-use and safety of a procedure are decisively influenced by imaging. Therefore, advances in this field are not only interesting for industry, engineers and dedicated imagers, but there are of major interest for the entire cardiology community and especially for the interventionalist. And further advances are needed: (i) Transoesophageal approaches with consecutive anaesthesia and possible complications are sub-optimal and should be avoided in an ideal world. (ii) Spatial and temporal resolution and acquisition time for all modalities requires further improvement. (iii) For faster orientation and procedure and accelerated learning curves, fusion imaging would be instrumental. (iv) Diagnostic performance requires better reproducibility, before, during and after the procedure. AI-based techniques should help to unify and simplify this. (v) On a future wish list and in a world of virtual reality and artificial intelligence, imaging instruments such as TEE or ICE should be maneuvered not by additional personnel, but by the interventionalist via robotic assistance directed by e.g. voice recognition. We are coming a long way from early x-rays and first echocardiography M-mode type of attempts, but there is still more to develop and discover and imaging will stay a rate-limiting and essential cornerstone of advances in interventional cardiology.

## Data Availability

This manuscript does not involve the collection of any data.
